# The Impact of Lower Extremity Skeletal Muscle Atrophy and Myosteatosis on Revascularization Outcomes in Patients with Peripheral Arterial Disease

**DOI:** 10.3390/jcm10173963

**Published:** 2021-08-31

**Authors:** Kirsten F. Ma, Stef Levolger, Issi R. Vedder, Mostafa El Moumni, Jean-Paul P. M. de Vries, Reinoud P. H. Bokkers, Alain R. Viddeleer

**Affiliations:** 1 Department of Surgery, Division of Vascular Surgery, University of Groningen, University Medical Center Groningen, 9700 RB Groningen, The Netherlands; j.p.p.m.de.vries@umcg.nl; 2 Department of Radiology, Medical Imaging Center, University of Groningen, University Medical Center Groningen, 9700 RB Groningen, The Netherlands; s.levolger@umcg.nl (S.L.); i.r.vedder@umcg.nl (I.R.V.); r.p.h.bokkers@umcg.nl (R.P.H.B.); a.r.viddeleer@umcg.nl (A.R.V.); 3 Department of Surgery, Division of Trauma Surgery, University of Groningen, University Medical Center Groningen, 9700 RB Groningen, The Netherlands; m.el.moumni@umcg.nl

**Keywords:** muscle atrophy, myosteatosis, peripheral arterial disease, lower extremity, revascularization

## Abstract

Background: This study investigated whether lower extremity muscle atrophy and myosteatosis in patients with peripheral arterial disease (PAD) are correlated to postoperative outcomes, such as reintervention or amputation-free survival. Methods: In this single-center retrospective cohort study of 462 patients treated for peripheral arterial disease scheduled for intervention, muscle mass and the presence of fattening of the lower extremity muscles were measured semiautomatically in a single computed tomography slice of the treated leg. Binary logistic regression models and Cox proportional hazards models were used to determine the effect of muscle atrophy and myosteatosis on reintervention and amputation. Results: Muscle atrophy and myosteatosis increased in PAD patients with Fontaine class IV compared with Fontaine class IIa. In PAD patients with muscle atrophy or myosteatosis, no association was found with the reintervention rate or reintervention-free survival, but an association was found with amputation-free survival, even after adjustment for patient-related, disease-severity, and comorbidities-related factors. Conclusion: Muscle atrophy and mysosteatosis increased in PAD patients with increasing disease severity. Lower extremity muscle atrophy and myosteatosis are associated with amputation rate and amputation-free survival in PAD patients. No association with reintervention rate or reintervention-free survival was found. Muscle atrophy and myosteatosis may serve as additional risk factors in decision making in the often frail vascular patient.

## 1. Introduction

Peripheral arterial disease (PAD) and chronic limb-threatening ischemia (CLTI) are associated with impaired quality of life and poor life expectancy [1]. Patients present with claudication, rest pain, and tissue loss [2]. These symptoms are caused by hypoperfusion of the limb tissue including muscles, which is caused by blockages in the arteries to the lower limbs [3]. In most PAD patients, atherosclerotic subintimal accumulation of lipids and fibrous material is the main cause of hypoperfusion of the limbs.

The lower extremity skeletal muscles in patients with PAD often demonstrate changes in morphology and function, characterized by increased fat infiltration, fibrosis, and decreased function [4,5]. Generalized loss of skeletal muscle mass, known as sarcopenia, is strongly associated with PAD and overall survival [6,7,8]. In lower extremity PAD, however, muscle changes in the limbs are more pronounced and increase with disease severity [9]. This is most likely caused by a combination of decreased physical activity and hypoperfusion-induced ischemia. In PAD-associated ischemia-reperfusion (IR) cycles, reactive oxygen species (ROS) production, mitochondrial dysfunction, and inflammation may be observed [10,11] as well as microcirculatory changes. Skeletal muscles are known to be particularly susceptible to these ischemic changes, with a short critical tissue ischemia time of just 3 h [11].

While focal atrophy and systemic sarcopenia are both associated with inflammation, the effects may be more pronounced within the diseased extremity itself, given the fact that the IR cycles and subsequent proteolytic and proinflammatory changes are expressed locally. Quantification of local muscle atrophy and intermuscular fat infiltration (myosteatosis) may potentially be candidate imaging markers reflecting the severity of IR disease and potential irreversibility of tissue damage and microcirculatory changes. It may have a strong association with disease-specific outcomes as well as treatment outcomes.

Thus, we hypothesized that muscle atrophy and myosteatosis of the lower extremity skeletal muscles are associated with postoperative outcomes, such as reintervention or amputation-free survival after lower leg revascularization procedures in patients with PAD. The aim of this study is to investigate the effect of muscle atrophy and myosteatosis on the risk of reintervention and amputation-free survival. In addition, we evaluated the association between disease severity and lower extremity skeletal muscle atrophy and myosteatosis.

## 2. Materials and Methods

This is a single-center retrospective cohort study of patients with peripheral arterial disease scheduled for an intervention of the lower limb between 1 January 2005 and 31 December 2017 at the University Medical Center Groningen (UMCG), a tertiary referral centre for vascular pathology located in the Northern region of The Netherlands. The Institutional Review Board reviewed the study (METc 2018/334). Individual informed consent was not required because studies involving a retrospective review, collection, and analysis of patient records do not fall under the scope of the Dutch Act on Medical Scientific Research involving Human Beings (WMO). For privacy, data were stored and analyzed pseudonymized.

### 2.1. Patient Selection

**Figure 1:**
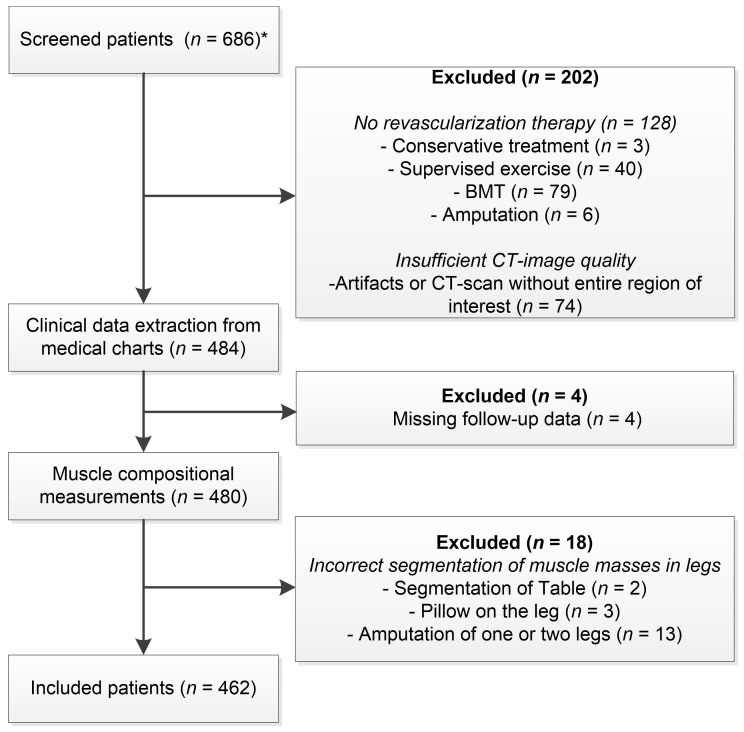
Workflow and selection process of included patients. (BMT) = best medical treatment, CT = computed tomography, * Patients screened for eligibility were retrieved from an existing dataset [8].

All patients referred by vascular surgeons for computed tomography (CT) angiography of the lower extremity arteries during the study period were considered for inclusion into this study. The patients were identified by querying the Picture Archiving and Communications System (PACS) of the UMCG. Patients diagnosed with Fontaine IIa to IV, aged > 18 years, undergoing endovascular therapy, surgery, or hybrid revascularization of the lower limb, and the availability of a diagnostic CT imaging scan within 1 year before revascularization were screened for inclusion (Figure 1). If a patient was scanned multiple times, only the last scan before the initial revascularization procedure was included. Exclusion criteria were a history of muscular disease, patients with acute ischemia, patients with a popliteal artery aneurysm, a CT scan without the entire area of interest, or a CT scan with severe artifacts. Patient characteristics, such as age, body mass index (BMI), sex, smoking status, type of intervention, comorbidities, and other risk factors, were extracted from the electronic medical records.

### 2.2. Muscle Imaging

Muscle atrophy and myosteatosis were defined by means of CT imaging. Images were acquired on a Siemens SOMATOM Definition (AS, Edge, Flash; Siemens Medical, Erlangen, Germany), SOMATOM Force (Siemens Medical, Erlangen, Germany), or Sensation (Siemens Medical, Erlangen, Germany). All scans were performed with intravenous contrast Iomeprol 350 (Iomeron) mgl/mL of 100 mL injected with a flow of 4 mL/s and scanned in the arterial phase. The images were acquired using a 512 × 512 scan matrix; slice thickness varied between 1 and 5 mm. All slices were stored in 16-bit Digital Imaging and Communications in Medicine (DICOM) format for further processing.

**Figure 2:**
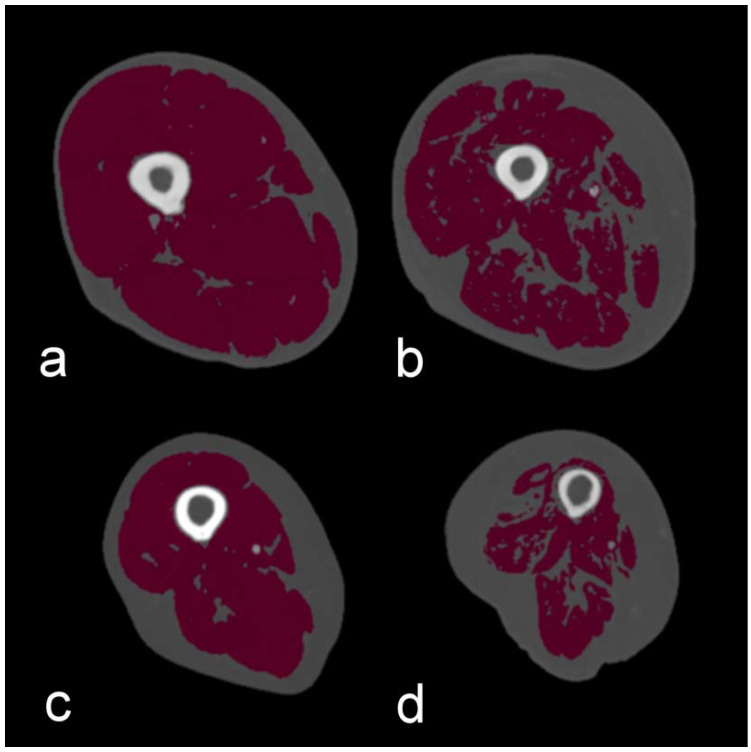
Cross-sectional skeletal muscle measurements of the lower extremity at 60% of the length of the femur, as seen from the knee. (**a**) No muscle atrophy and non-myosteatotic and (**b**) No muscle atrophy, but myosteatotic (**c**) Muscle atrophy, but non-myosteatotic. (**d**) Muscle atrophy and myosteatotic.

The muscle mass and presence of fat infiltration in the lower extremity skeletal muscles were measured semiautomatically by means of in-house developed software (SarcoMeas Extremity, v1.1; UMCG, Groningen, The Netherlands). For this, the area and Hounsfield units (HU) of the treated lower extremity skeletal muscles were measured on one CT slice of the upper leg, at 60% of the length of the femur as seen from the knee, according to a previously published study [12] (Figure 2). If both legs were treated within one revascularization procedure, only 1 leg was randomly selected for analysis. Muscle mass was automatically segmented from the CT scans based on tissue attenuation and morphology, excluding skin and bone. The threshold used for the segmentation of muscle tissue was in the range of −29 to +150 HU [13].

To define muscle atrophy in the legs, the muscle area measured on the cross-sectional slices of the treated leg was normalized to the patient’s height by dividing the segmented leg muscle area by the squared patient height to form the lower extremity skeletal muscle index (LESMI) in cm^2^/m^2^. Cutoff values for muscle atrophy were determined based on sex. For myosteatosis, the mean HU value of the segmented muscle voxels was determined from the cross-sectional slice of the treated leg. Cutoff values for myosteatosis were determined for patients with a BMI of <25 or >25 kg/m^2^. 

### 2.3. Outcome Variables and Follow-Up

The primary outcomes of interest were amputation and reintervention rates of the treated lower extremity. At the end of follow-up the end-points reintervention-free survival and amputation-free survival were determined. Reintervention was defined as any reintervention of the treated leg. Amputation rates included minor and major amputations of the lower extremity after revascularization. Follow-up data until 1 October 2018 were collected from the electronic medical records. Secondary outcome was the effect of increasing disease severity upon CT imaging parameters as LESMI and muscle HU.

### 2.4. Statistical Analysis

Statistical analyses were performed with SPSS 23 software (IBM Corp, Armonk, NY, USA). A *p* value of <0.05 was considered to be statistically significant. Descriptive statistics are presented as mean ± standard deviation (SD) for normally distributed data or as medians with interquartile ranges (IQR) otherwise.

Cutoff values for muscle atrophy and myosteatosis in the legs were determined with optimal stratification and receiver operating characteristic curve analysis. Optimal stratification is a technique, based on log-rank statistics, to determine at which cutoff values the most significant difference would occur with regard to a binary or time-to-event outcome [8,14,15]. Between-group changes were assessed using one-way analysis of variance (ANOVA). Binary logistic regression models were used to analyse the association of muscle atrophy and myosteatosis in the legs with reintervention and amputation after revascularization procedures.

Models were used to control for different confounders. Interaction terms for chronic pulmonary disease were tested [16,17,18]. Four models were determined. The first model investigated the effect of muscle atrophy or myosteatosis on reintervention or amputation without controlling for other prognostic factors. The second model controlled for patient-related factors (i.e., age, BMI, smoking status). The third model controlled for patient-related and disease-related factors (i.e., type of intervention and Fontaine class) [19,20], and the fourth model controlled for patient-, disease-, and comorbidities-related factors (i.e., hypertension, hypercholesterolemia, coronary artery disease, hemodialysis, and ischemic stroke) [19,21]. For the amputation regression models, sex, chronic obstructive pulmonary disease (COPD), and type 2 diabetes were added [19,22,23].

Multivariable imputation by chained equations was used for multiply imputation of missing data [24]. The number of imputations was determined according to the two-stage procedure as recommended by Von Hippel [25]. A total of 10 different imputed data sets were constructed. Parameters, with their standard errors, were estimated with binary logistic regression and Cox regression applied to each data set separately, and pooled using Rubin’s rule [26]. Apart from the variables of interest, the Nelson–Aalen estimator was calculated and also included in the imputation algorithm [27].

The ascertained groups and potential prognostic factors were analyzed using a univariable Cox proportional hazards model with the same models stated above. The included number of independent variables was based on 10 events per variable, a widely used minimal criterion [28]. Models were controlled for confounders, and interaction terms were tested. Kaplan–Meier method with log-rank testing was used for amputation-free survival curves performed with MATLAB R2019a software (The Math Works, Inc., Natick, MA, USA).

## 3. Results

The study included 462 patients with PAD (Figure 1). The median follow-up time was 2.4 (IQR, 1.4–3.8) years. The mean time between CT scan and intervention was 60.6 ± 64.8 days. The patient characteristics are summarized in Table 1. Only 438 patients were classified with a smoking status and 424 patients with Fontaine class due to a lack of classification within medical records. Most patients were Fontaine class IIb (32.0%) and class IV (30.1%). Patients (*n* = 462) had a high prevalence of typical vascular comorbidities such as hypertension (66.5%), hypercholesterolemia (82.3%), and coronary arterial disease (64.3%).

The mean LESMI of the treated leg was 35.2 ± 9.0 cm^2^/m^2^ in men (*n* = 273) and 29.1 ± 8.7 cm^2^/m^2^ in women (*n* = 189). Patients with Fontaine class IV (*n* = 139) had a lower LESMI of 28.4 ± 8.9 cm^2^/m^2^ compared with 42 patients with Fontaine class IIa (36.2 ± 7.1 cm^2^/m^2^), Table 2. The average BMI-specific myosteatosis value for the treated leg for PAD patients with a BMI < 25 kg/m^2^ (*n* = 215) was 45.3 ± 8.6 mean HU, which differed between men (45.9 ± 7.2 mean HU) and women (44.5 ± 10.1 mean HU). For patients with a BMI > 25 kg/m^2^ (*n* = 247), the mean HU was 42.4 ± 8.4 (43.3 ± 7.3 for men and 41.1 ± 9.8 for women). The mean HU decreased from 49.7 ± 6.9 for Fontaine class IIa patients (*n* = 42) to 39.3 ± 9.1 for Fontaine class IV patients (*n* = 139), Table 2.

Sex-specific LESMI cutoff values for muscle atrophy in the legs were 32.5 cm^2^/m^2^ for men and 24.5 cm^2^/m^2^ for women, which resulted in 153 of 462 PAD patients (33.1%) who had muscle atrophy in the legs. Calculated BMI-specific cutoff mean HU values for myosteatosis in the legs were 44.0 for patients with a BMI < 25 kg/m^2^, and 41.0 for patients with a BMI > 25 kg/m^2^. This resulted in 180 of 462 patients (39.0%) who were diagnosed with myosteatosis of the legs.

After a mean follow-up of 36 months, the reintervention rate after the initial revascularization was 19.8% (*n* = 61) within the nonmuscle-atrophic group, 14.9% (*n* = 23) in the muscle-atrophic group, 18.8% (*n* = 53) within the non-myosteatotic group, and 17.2% (*n* = 31) in the myosteatotic group. Muscle atrophy and myosteatosis of the lower extremity skeletal muscle were not associated with reintervention (Table 3).

The amputation rate after the initial revascularization was 9.4% (*n* = 29) for the nonmuscle-atrophic group vs. 24.0% (*n* = 37) in the muscle-atrophic group, and 9.6% (*n* = 27) for the non-myosteatotic group vs. 21.7% (*n* = 39) in the myosteatotic group. The impact of muscle atrophy and myosteatosis of the lower extremity skeletal muscle on amputation is provided in Table 4. Model 4, controlled for patient-, disease-, and comorbidities-related factors, shows that PAD patients with muscle atrophy have an odds ratio of 2.351 (95% confidence interval (CI), 1.157–4.776; *p* = 0.018) for amputation. PAD patients with myosteatosis have an odds ratio of 2.142 (95% CI, 1.114–4.116; *p* = 0.022) for an amputation. COPD did not change the relationship of muscle atrophy or myosteatosis with reintervention or amputation.

The multivariable Cox proportional hazards model controlled for patient-, disease-, and comorbidities-related factors for muscle atrophy and myosteatosis measured for reintervention and amputation-free survival is summarized in Table 5 and Table 6. For reintervention-free survival, the multivariable Cox proportional hazards model showed a hazard ratio (HR) of 0.871 (95% CI, 0.508–1.495; *p* = 0.616) for PAD patients with muscle atrophy and an HR of 1.113 (95% CI, 0.685–1.808; *p* = 0.667) for PAD patients with myosteatosis. For amputation-free survival, the multivariable Cox proportional hazards model showed an HR of 2.017 (95% CI, 1.108–3.671; *p* = 0.022) for PAD patients with muscle atrophy and an HR of 2.116 (95% CI, 1.229–3.645; *p* = 0.007) for patients with myosteatosis. COPD did not change the relationship of muscle atrophy or myosteatosis on reintervention-free survival or amputation-free survival. Univariable and multivariable Cox proportional hazards models (model 1–model 3) for muscle atrophy and myosteatosis measured in the legs for reintervention-free survival and amputation-free survival are shown in the Supplementary Material Section (Tables S1–S6).

**Figure 3:**
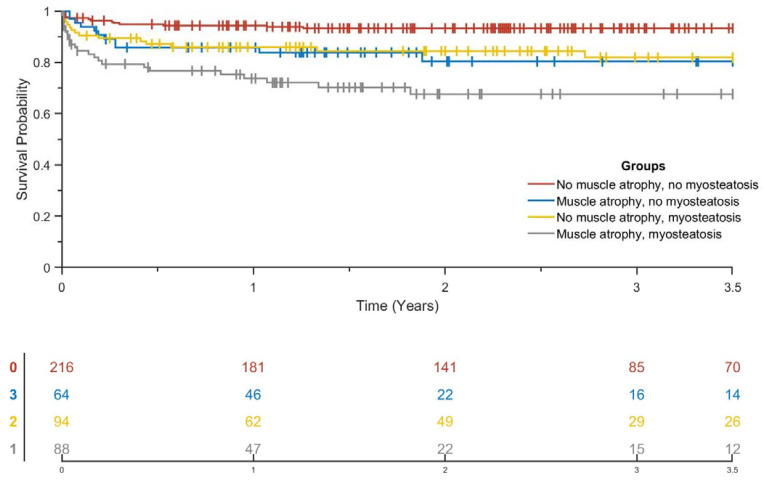
Kaplan–Meier amputation-free survival curves of patients with or without different forms of muscle depletion.

The Kaplan–Meier analysis for amputation-free survival showed that patients with combined muscle atrophy and myosteatosis have a 3-year amputation-free survival of 67.6% compared to 93.3% for patients without muscle atrophy and myosteatosis (Figure 3). Patients without muscle atrophy, with myosteatosis have a 3-year amputation-free survival of 81.9% compared with 80.3% for patients with muscle atrophy but without myosteatosis (Figure 3).

## 4. Discussion

The results of this study show that the occurrence of muscle atrophy and myosteatosis of lower extremity skeletal muscles are not associated with higher reintervention rates but are associated with amputation rates after revascularization therapy in patients with PAD. The association with reduced amputation-free survival remained statistically significant after adjustment for patient-related, disease-severity, and comorbidities-related factors.

Previous studies have found that PAD is associated with decreased muscle cross-sectional area. An early study by Regenstein et al. [4] found a significant reduction in cross-sectional muscle area in patients with intermittent claudication compared with the non-symptomatic contralateral legs. Their study also reported an ipsilateral reduction in gastrocnemius strength and endurance, anterior tibial strength, and histopathologic changes in muscle fiber characteristics.

A reduced ankle-brachial index, which is a marker for decreased limb perfusion, is also associated with lower calf muscle cross-sectional area and higher calf muscle percentage fat [9]. These changes have been found to be associated with mobility loss at follow-up [29]. IR injury is thought to contribute to this low extremity muscle atrophy and myosteatotic change in PAD patients [5].

Skeletal muscle mass is known to be particularly prone to ischemic changes, having the shortest critical tissue ischemia times in the extremities, with rapid onset of necrosis extending up to 90% of affected muscle tissue at 5 h [11]. In this study, we observed the lowest amputation-free survival rates in patients with the worst imaging muscle characteristics (i.e., concomitant muscle atrophy and myosteatosis). Although this study lacks a histopathologic correlate, we speculate that these imaging characteristics reflect more profound IR injury and its detrimental effects, including associated microvascular changes, ultimately leading to failure of provided endovascular, surgical, or hybrid therapy in these patients and necessitating amputation.

Reintervention after revascularization was not associated with muscle atrophy or myosteatosis. This is possibly explained due to the multifactorial nature of the reintervention requirement. First, the high rate of amputation, in particular in the patient group affected by both muscle atrophy and myosteatosis, could lead to survival bias, wherein the patient group not requiring amputation has favorable clinical characteristics and status of vascular disease, which protected them from not only amputation but also reintervention. The remainder of patients requiring reintervention may find similar grounds for failure as patients without muscle atrophy or myosteatosis, including technical failure or possible suboptimal compliance with medical treatment, continued smoking, and clopidogrel resistance [30].

Medical management in PAD is primarily aimed at risk factor reduction, supervised exercise programs, and revascularization therapy for ischemic rest pain or tissue loss. Given revascularization therapy is not always effective and may incur significant morbidity and mortality, guidelines have been introduced to help decide whether treatment may be successful. The current study indicates the lower extremity muscle measurements are an imaging marker that could be used in clinical decision making, potentially aiding in the selection of patients in whom an elevated amputation risk along with present comorbidity and biological age may warrant primary amputation.

Future studies may consider the use of CT perfusion, which may allow for quantification of the perfusion status and microcirculation of the foot [31,32], to further narrow the group of patients at high risk for amputation. Furthermore, prospective studies are required to determine whether earlier revascularization based on muscle atrophy and myosteatotic change in follow-up imaging, rather than conventional criteria alone, may improve amputation-free survival rates in patients with PAD.

Moreover, there is an interest for pharmaceutical intervention strategies, which may alleviate effects of oxidative stress. For instance, cocoa, which contains phenolic antioxidants and flavonoids, including epicatechin [33], was suggested to improve mitochondrial activity, increase capillary density, improve calf muscle perfusion, and improve the 6-min walk distance in a recent double-blind, pilot randomized controlled trial [34]. However, as concluded in a review by Giannopoulous et al. [35], clinical results of such antioxidant therapies in PAD have thus far been dissatisfactory. There is interest in the development of pharmacologic approaches directly modulating mitochondrial function, with promising results in preclinical studies; however, translation to clinical trials is slow [36].

This study has several limitations. First, the scans used in this study were acquired in the arterial phase. Despite the contrast bolus being predominantly in the large vessels and a threshold being applied to extract intravascular signal, it may potentially have caused higher density measurements in the muscles.

Another limitation is that we were not able to assess muscle strength with these CT measurements. Including muscle strength would improve muscle assessment for systematic muscle failure, according to the European Working Group on Sarcopenia in Older People (EWGSOP) 2 [37].

This study lacks a comparison to the asymptomatic leg, which was not possible due to missing Fontaine classifications of the contralateral leg. This would be of great interest for future studies.

Lastly, the retrospective nature of the current study may have led to selection bias. National guidelines were, however, followed for diagnosis and treatment, and patients are routinely discussed in a multidisciplinary vascular board consisting of vascular surgeons and interventional radiologists before treatment selection.

In conclusion, muscle atrophy and myosteatosis decreased in PAD patients with an increase in disease severity. There was no association with reintervention rate or reintervention-free survival. However, lower extremity skeletal muscle atrophy and myosteatosis are associated with amputation rate and a reduction in amputation-free survival in patients with PAD and may serve as additional risk factors in decision making in the often frail vascular patients.

## Figures and Tables

**Table 1:** Patient characteristics.

	*n* = 462
Age (year)	65.2 ± 11
BMI (kg/m^2^)	25.9 ± 4.7
	*n* = 438
Smoking	273 (59.1%)
Sex	
- Female	189 (40.9%)
- Male	273 (59.1%)
Type of intervention	
- Surgical	137 (29.7%)
- Endovascular	268 (58.0%)
- Hybrid	57 (12.3%)
COPD	121 (26.2%)
Type 2 diabetes	139 (30.1%)
Hypertension	307 (66.5%)
Hypercholesterolemia	380 (82.3%)
Coronary artery disease	297 (64.3%)
Hemodialysis	21 (4.5%)
Ischemic stroke	35 (7.69%)
Classification	*n* = 424
- Fontaine IIa	42 (9.1%)
- Fontaine IIb	148 (32.0%)
- Fontaine III	95 (20.6%)
- Fontaine IV	139 (30.1%)

BMI = body mass index, COPD = chronic obstructive pulmonary disease.

**Table 2:** LESMI and Mean HU of treated legs of PAD patients divided by disease severity.

	Fontaine Classification IIa	Fontaine Classification IIb	Fontaine Classification III	Fontaine Classification IV	*p*-Value
Total *n* = 424	*n* = 42	*n* = 148	*n* = 95	*n* = 139	
LESMI at cross-sectional slice at 60% (cm^2^/m^2^)	36.2 (±7.1)	35.9 (±8.8)	31.8 (±8.7)	28.4 (±8.9)	<0.001
Mean HU at cross-sectional slice at 60%	49.7 (±6.9)	45.6 (±6.7)	44.8 (±8.4)	39.2 (±9.1)	<0.001

LESMI = lower extremity skeletal muscle index (cm^2^/m^2^), HU = Hounsfield units. *p* value stated in bold indicates statistical significance of <0.05.

**Table 3:** Binary logistic regression for the impact of muscle atrophy or myosteatosis on reintervention in four models controlled for different risk factors.

	Muscle Atrophy Leg			Myosteatosis Leg		
	Odds Ratio	95% CI	*p*-Value	Odds Ratio	95% CI	*p*-Value
Model 1	0.711	0.421–1.201	0.202	0.899	0.551–1.466	0.669
Model 2	0.866	0.648–1.157	0.619	1.061	0.630–1.786	0.823
Model 3	0.776	0.433–1.391	0.394	0.963	0.563–1.649	0.891
Model 4	0.743	0.407–1.357	0.333	0.958	0.558–1.645	0.877

Model 1 = Muscle atrophy or myosteatosis on reintervention. Model 2 = Muscle atrophy or myosteatosis on reintervention controlled for patient-related factors (i.e., age, BMI, and smoking status). Model 3 = Muscle atrophy or myosteatosis on reintervention controlled for both patient-related and disease-related factors (i.e., type of intervention and Fontaine class). Model 4 = Muscle atrophy or myosteatosis on reintervention controlled for patient-related, disease-related, and comorbidities-related factors (i.e., hypertension, hypercholesterolemia, coronary artery disease, hemodialysis, and ischemic stroke). CI = Confidence interval.

**Table 4:** Binary logistic regression for the impact of muscle atrophy or myosteatosis on amputation in four models controlled for different risk factors.

	Muscle Atrophy Leg			Myosteatosis Leg		
	Odds Ratio	95% CI	*p*-Value	Odds Ratio	95% CI	*p*-Value
Model 1	3.042	1.787–5.179	**<0.001**	2.612	1.534–4.447	**<0.001**
Model 2	4.080	2.430–6.852	**<0.001**	3.274	1.814–5.908	**<0.001**
Model 3	2.949	1.501–5.790	**0.002**	2.288	1.209–4.329	**0.011**
Model 4	2.351	1.157–4.776	**0.018**	2.142	1.114–4.116	**0.022**

Model 1 = Muscle atrophy or myosteatosis on amputation. Model 2 = Muscle atrophy or myosteatosis on amputation controlled for patient-related factors (i.e., age, BMI, sex, smoking status, COPD, DM type II). Model 3 = Muscle atrophy or myosteatosis on amputation controlled for both patient-related and disease-related factors (i.e., type of intervention and Fontaine class). Model 4 = Muscle atrophy or myosteatosis on amputation controlled for patient-related, disease-related, and comorbidities-related factors (i.e., hypertension, hypercholesterolemia, coronary artery disease, hemodialysis, and ischemic stroke). CI = confidence interval. *p* value stated in bold indicates statistical significance of <0.05.

**Table 5:** Multivariable Cox proportional hazards model for muscle atrophy and myosteatosis for reintervention free survival in PAD patients.

Variables	Muscle Atrophy Leg			Myosteatosis Leg		
	HR	95% CI	*p*-Value	HR	95% CI	*p*-Value
Muscle atrophy Legs	0.871	0.508–1.495	0.616			
Myosteatosis Legs				1.113	0.685–1.808	0.667
Age (y)	0.980	0.959–1.001	0.066	0.978	0.957–1.000	**0.049**
BMI (kg/m^2^)	1.026	0.974–1.081	0.331	1.030	0.981–1.081	0.230
Smoking	0.862	0.520–1.430	0.566	0.870	0.525–1.439	0.586
Type of intervention						
Surgical	Ref.			Ref.		
Endovascular	1.023	0.630–1.661	0.925	1.021	0.629–1.657	0.933
Hybrid	1.109	0.529–2.328	0.784	1.124	0.536–2.357	0.757
Fontaine class						
IIa & IIb	Ref.			Ref.		
III & IV	1.716	1.056–2.789	**0.029**	1.648	1.012–2.686	**0.045**
Hypertension	1.001	0.612–1.637	0.998	1.012	0.618–1.656	0.963
Hypercholesterolemia	1.146	0.618–2.125	0.665	1.157	0.624–2.144	0.644
Coronary artery disease	1.094	0.688–1.740	0.705	1.075	0.677–1.706	0.760
Hemodialysis	1.896	0.718–5.005	0.197	1.808	0.692–4.724	0.227
Ischemic stroke	1.055	0.451–2.466	0.902	1.019	0.437–2.377	0.965

Model 4 = Controlled for patient-related, disease-related, and comorbidities-related factors (i.e., hypertension, hypercholesterolemia, coronary artery disease, hemodialysis, and ischemic stroke). CI = confidence interval. *p* value stated in bold indicates statistical significance of <0.05.

**Table 6:** Multivariable Cox proportional hazards model for muscle atrophy and myosteatosis for amputation-free survival in PAD patients.

Variables	Muscle Atrophy Leg			Myosteatosis Leg		
	HR	95% CI	*p*-Value	HR	95% CI	*p*-Value
Muscle atrophy Legs	2.017	1.108–3.671	**0.022**			
Myosteatosis Legs				2.116	1.229–3.645	**0.007**
Age (y)	0.986	0.963–1.009	0.224	0.983	0.959–1.007	0.162
Sex						
Male	Ref.			Ref.		
Female	0.998	0.595–1.675	0.995	0.915	0.544–1.538	0.738
BMI (kg/m^2^)	1.051	0.990–1.115	0.101	1.024	0.969–1.082	0.395
Smoking	1.134	0.635–2.027	0.670	1.221	0.678–2.200	0.506
Type 2 diabetes	1.680	0.953–2.963	0.073	1.911	1.102–3.312	**0.021**
COPD	0.479	0.242–0.945	**0.034**	0.451	0.227–0.896	**0.023**
Type of intervention						
Surgical	Ref.			Ref.		
Endovascular	0.802	0.448–1.436	0.457	0.778	0.440–1.377	0.388
Hybrid	1.146	0.558–2.354	0.710	1.105	0.539–2.262	0.786
Fontaine class						
IIa & IIb	Ref.					
III & IV	17.729	5.439–57.791	**<0.001**	18.134	5.554–59.205	**<0.001**
Hypertension	0.842	0.484–1.462	0.541	0.841	0.488–1.452	0.535
Hypercholesterolemia	0.714	0.376–1.356	0.304	0.619	0.330–1.162	0.136
Coronary artery disease	0.937	0.542–1.620	0.816	0.886	0.515–1.526	0.663
Hemodialysis	3.256	1.519–6.981	**0.002**	4.017	1.924–8.387	**<0.001**
Ischemic stroke	0.821	0.314–2.149	0.688	0.939	0.359–2.458	0.898

Model 4 = Controlled for patient-related, disease-related, and comorbidities-related factors (i.e., hypertension, hypercholesterolemia, coronary artery disease, hemodialysis, and ischemic stroke). CI = confidence interval, BMI = body mass index, COPD = chronic obstructive pulmonary disease. *p* value stated in bold indicates statistical significance of <0.05.

## Data Availability

The data presented in this study are available on request from the corresponding author. The data are not publicly available due to privacy and ethical reasons.

## Supplementary Materials

The following are available online at https://www.mdpi.com/article/10.3390/jcm10173963/s1, Table S1: Univariable Cox proportional hazards model for reintervention free survival in PAD patients, Table S2: Multivariable Cox proportional hazards model for muscle atrophy and myosteatosis for reintervention free survival in PAD patients, Table S3: Multivariable Cox proportional hazards model for muscle atrophy and myosteatosis for reintervention free survival in PAD patients, Table S4: Univariable Cox proportional hazards model for amputation-free survival in PAD patients, Table S5: Multivariable Cox proportional hazards model for muscle atrophy and myosteatosis for amputation-free survival in PAD patients, Table S6: Multivariable Cox proportional hazards model for muscle atrophy and myosteatosis for amputation-free survival in PAD patients.
