# Supplementary material for: The Impact of Lower Extremity Skeletal Muscle Atrophy and Myosteatosis on Revascularization Outcomes in Patients with Peripheral Arterial Disease
Source: J Clin Med. 2021 Aug 31;10(17):3963. doi: 10.3390/jcm10173963 (PMC8432022; doi:10.3390/jcm10173963)
Supplement: Supplementary file 1 [file jcm-10-03963-s001.zip › jcm-1280890-supplementary.pdf]

Supplementary Tables

Supplementary Table 1. Univariable Cox proportional hazards model for reintervention free survival in PAD patients

Variables	Univariable analysis Model 1		
	HR	95% CI	*p* value
Muscle atrophy Legs	0.889	0.696-1.136	0.632
Myosteatosis Legs	1.163	0.927-1.458	0.506
Age (y)	0.986	0.977-0.996	0.162
BMI (kg/m^2)	1.030	0.983-1.079	0.215
Smoking	0.945	0.591-1.512	0.813
Type of intervention			
Surgical	Ref		
Endovascular	1.003	0.787-1.280	0.989
Hybrid	1.151	0.782-1.695	0.705
Fontaine class			
IIa & IIb	Ref		
III & IV	1.584	1.006-2.496	**0.047**
Hypertension	0.964	0.766-1.213	0.873
Hypercholesterolemia	1.146	0.847-1.550	0.648
Coronary artery disease	1.118	0.893-1.400	0.620
Haemodialysis	2.247	0.904-5.585	0.081
Ischemic stroke	1.058	0.693-1.617	0.894

CI = confidence interval, BMI = body mass index.

Supplementary Table 2. Multivariable Cox proportional hazards model for muscle atrophy and myosteatosis for reintervention free survival in PAD patients

Variables	Multivariable analysis Model 2 muscle atrophy			Multivariable analysis Model 2 myosteatosis		
	HR	95% CI	*p* value	HR	95% CI	*p* value
Muscle atrophy legs	1.044	0.630-1.730	0.870			
Myosteatosis legs				1.269	0.794-2.029	0.320
Age (y)	0.984	0.964-1.004	0.117	0.981	0.961-1.002	0.076
BMI (kg/m^2)	1.032	0.982-1.084	0.218	1.028	0.981-1.078	0.247
Smoking	0.893	0.545-1.462	0.653	0.900	0.550-1.474	0.675

Model 2 = Controlled for patient-related factors (i.e., age, BMI, and smoking status). CI = confidence interval. *P* value stated in bold indicates statistical significance of <0.05.

Supplementary Table 3. Multivariable Cox proportional hazards model for muscle atrophy and myosteatosis for reintervention free survival in PAD patients

Variables	Multivariable analysis Model 3 muscle atrophy			Multivariable analysis Model 3 myosteatosis		
	HR	95% CI	*p*-value	HR	95% CI	*p* value
Muscle atrophy legs	0.922	0.546-1.555	0.760			
Myosteatosis legs				1.134	0.701-1.833	0.608
Age (y)	0.980	0.960-1.000	**0.049**	0.978	0.958-0.999	**0.037**
BMI (kg/m^2)	1.032	0.981-1.085	0.223	1.033	0.986-1.083	0.173
Smoking	0.848	0.517-1.392	0.514	0.855	0.522-1.402	0.535
Type of intervention						

Surgical	Ref			Ref		
Endovascular	1.007	0.623-1.627	0.979	1.005	0.622-1.625	0.983
Hybrid	1.120	0.536-2.341	0.764	1.128	0.540-2.357	0.749
Fontaine class						
IIa & IIb	Ref			Ref		
III & IV	1.766	1.097-2.843	**0.019**	1.703	1.056-2.749	**0.029**

Model 3 = Controlled for both patient-related and disease-related factors (i.e., type of intervention and Fontaine class). CI = confidence interval. *p* value stated in bold indicates statistical significance of <0.05.

Supplementary Table 4. Univariable Cox proportional hazards model for amputation free survival in PAD patients

Variables	Univariable analysis Model 1		
	HR	95% CI	*p*-value
Muscle atrophy Legs	2.970	1.824-4.835	**<0.001**
Myosteatosis Legs	2.641	1.616-4.318	**<0.001**
Age (y)	1.002	0.979-1.024	0.893
Sex			
Male	Ref		
Female	1.185	0.728-1.927	0.495
BMI (kg/m^2)	1.008	0.957-1.062	0.765
Smoking	0.892	0.581-1.603	0.965
Type 2 diabetes	1.806	1.108-2.943	**0.018**
COPD	0.539	0.282-1.029	0.061
Type of intervention			
Surgical	Ref		
Endovascular	0.760	0.440-1.311	0.323
Hybrid	1.383	0.683-2.802	0.368
Fontaine class			
IIa & IIb	Ref		
III & IV	19.744	6.225-62.625	**<0.001**
Hypertension	0.803	0.489-1.321	0.388
Hypercholesterolemia	0.638	0.364-1.121	0.118
Coronary artery disease	1.093	0.664-1.797	0.727
Hemodialysis	7.294	3.779-14.078	**<0.001**
Ischemic stroke	1.033	0.415-2.570	0.945

CI = confidence interval, BMI = body mass index, COPD = chronic obstructive pulmonary disease. *p* value stated in bold indicates statistical significance of <0.05.

Supplementary Table 5. Multivariable Cox proportional hazards model for muscle atrophy and myosteatosis for amputation free survival in PAD patients

Variables	Multivariable analysis Model 2 muscle atrophy			Multivariable analysis Model 2 myosteatosis		
	HR	95% CI	*p*-value	HR	95% CI	*p* value
Muscle atrophy legs	3.803	2.470-5.857	**<0.001**			
Myosteatosis legs				3.085	1.826-5.210	**<0.001**
Age (y)	0.992	0.971-1.014	0.469	0.990	0.967-1.014	0.414
Sex						
Male	Ref			Ref		

Female	1.221	0.749-1.991	0.424	1.133	0.695-1.849	0.617
BMI (kg/m^2)	1.047	0.989-1.107	0.115	0.987	0.937-1.039	0.607
Smoking	1.041	0.602-1.802	0.885	1.243	0.706-2.190	0.451
Type 2 diabetes	1.448	0.892-2.351	0.162	1.720	1.036-2.856	**0.036**
COPD	0.463	0.239-0.897	**0.023**	0.426	0.219-0.830	**0.012**

Model 2 = Controlled for patient-related factors (i.e., age, BMI, gender, and smoking status, DM type 2, and COPD). CI = confidence interval, BMI = body mass index, COPD = chronic obstructive pulmonary disease. *p* value stated in bold indicates statistical significance of <0.05.

Supplementary Table 6. Multivariable Cox proportional hazards model for muscle atrophy and myosteatosis for amputation free survival in PAD patients

Variables	Multivariable analysis Model 3 muscle atrophy			Multivariable analysis Model 3 myosteatosis		
	HR	95% CI	*p* value	HR	95% CI	*p*value
Muscle atrophy legs	2.627	1.496-4.612	**0.001**			
Myosteatosis legs				2.191	1.278-3.759	**0.004**
Age (y)	0.979	0.957-1.001	0.067	0.977	0.953-1.001	0.059
Gender						
Male	Ref			Ref		
Female	1.096	0.668-1.800	0.716	0.984	0.597-1.621	0.949
BMI (kg/m^2)	1.053	0.994-1.116	0.077	1.009	0.958-1.064	0.732
Smoking	1.016	0.580-1.779	0.957	1.125	0.633-1.999	0.688
Type 2 diabetes	1.448	0.834-2.515	0.189	1.778	1.047-3.018	**0.033**
COPD	0.436	0.223-0.853	**0.015**	0.414	0.210-0.816	**0.011**
Type of intervention						
Surgical	Ref			Ref		
Endovascular	0.856	0.484-1.513	0.592	0.839	0.477-1.475	0.542
Hybrid	1.129	0.552-2.309	0.740	1.109	0.544-2.262	0.775
Fontaine class						
IIa & IIb	Ref			Ref		
III & IV	18.597	5.727-60.386	**<0.001**	19.152	5.889-62.277	**<0.001**

Model 3 = Controlled for both patient-related and disease-related factors (i.e., type of intervention and Fontaine class). CI = confidence interval, BMI = body mass index, COPD = chronic obstructive pulmonary disease. *p* value stated in bold indicates statistical significance of <0.05.
